# Electrochemical Upgrading of Waste Polylactic Acid Plastic for the Coproduction of C_2_ Chemicals and Green Hydrogen

**DOI:** 10.3390/molecules29225323

**Published:** 2024-11-12

**Authors:** Daili Xiang, Kexin Zhou, Jiahui Huang, Qing Kang, Hao Li, Yuhui Duan, Jialei Du, Hong Liu

**Affiliations:** 1 Institute for Advanced Interdisciplinary Research (iAIR), Collaborative Innovation Center of Technology and Equipment for Biological Diagnosis and Therapy in Universities of Shandong, University of Jinan, Jinan 250022, China; 2 School of Chemistry and Chemical Engineering, University of Jinan, Jinan 250022, China; 3 Department of Chemistry, National University of Singapore, 3 Science Drive 3, Singapore 117543, Singapore; 4 State Key Laboratory of Crystal Materials, Shandong University, Jinan 250100, China

**Keywords:** hybrid water electrolysis, polylactic acid plastic, *Saccharomycetes*, lactate oxidation, hydrogen production

## Abstract

Tandem alkali-catalyzed hydrolysis and alkaline electrolysis have gradually become appealing avenues for the reformation of polyester plastics into high-value-added chemicals and green hydrogen with remarkable environmental and economic benefits. In this study, an electrochemical upcycling strategy was developed for the electrocatalytic oxidation of polylactic acid (PLA) hydrolysate into valued C_2_ chemicals (i.e., acetate) and hydrogen fuel using N, P-doped CuO_x_ nanowires (NW) supported on nickel foam (NF) as the electrocatalyst. This 3D well-integrated catalyst was easily prepared from a Cu(OH)_2_ NW/NF precursor with *Saccharomycetes* as a green and safe P and N source. The electrocatalyst can efficiently catalyze the lactate monomer derived from the hydrolysis of PLA waste to acetate with high selectivity and exhibits a lower onset potential for the lactate oxidation reaction (LOR) than for water oxidation, saving 224 mV to deliver a current density of 30 mA/cm^2^. The experimental results reveal that the plausible pathway of the LOR on these CuO_x_ NW involves oxidation and subsequent decarboxylation. Divalent copper species have been verified to be active sites for LOR via in situ Raman spectroscopy.

## 1. Introduction

Water splitting, powered by electricity generated from renewable but intermittent energy sources (e.g., solar, wind, and hydropower), has been extensively explored for massive H_2_ production with low CO_x_ emissions [1,2,3,4]. Conventional water electrolysis comprises two half-reactions: the anodic oxygen evolution reaction (OER) and cathodic hydrogen evolution reaction (HER). The OER donates abundant reductive equivalents (e^−^/H^+^) to the HER, and from this perspective, renewable water acts as a reservoir of electrons and protons [5]. However, OER is kinetically unfavorable, and a large potential value exceeding 1.23 V is regularly required to drive water splitting. Furthermore, these two reactions are strictly coupled, indicating that O_2_ and H_2_ are simultaneously generated. As a result, the purity of H_2_ may be influenced, and even a hazardous H_2_/O_2_ mixed gas could be formed owing to the inevitable gas crossover. Ironically, O_2_ is regarded as an insignificant product because it is widely present in air. As the cathode product, the target H_2_ gas actually burdens the cost of the two electrode reactions. In addition, the co-existence of O_2_, H_2_, and electrocatalysts would generate harmful reactive oxygen species, which would accelerate the deactivation of the proton exchange membrane. Thus, the OER side remains a key bottleneck hindering the water electrolysis technology [6,7,8,9].

Hybrid water electrolysis (H_2_O + AF_red_ → H_2_ + AF_ox_, where AF represents the anode feedstock) has been recently proposed to ameliorate the two main problems of elevated energy consumption and low added value of anode products, which are inherent in the conventional process [6]. The nucleophilic AFs are easily oxidized before water molecules; hence, the OER is replaced by oxidation reactions (AF_red_ → AF_ox_ + ne^−^) that are thermodynamically and economically more favorable [6]. Diverse value-added chemicals, but not the unrequired O_2,_ can be selectively generated at the anode with lower voltage inputs, while pure H_2_ is efficiently co-produced at the cathode (2H^+^ + 2e^−^ → H_2_). The potentials of the two electrodes are fully exploited, resulting in an optimum overall Faradaic efficiency of approximately 200%. The careful selection of AFs has been demonstrated to be vital for developing hybrid water splitting to maximize the economic returns of energy investment. These AFs are often characterized by their easy availability, low cost, rich reserves, high water solubility, abundant hydrogen content, large appreciation space (from AF_red_ to AF_ox_), and oxidation potentials lower than those of the OER. Recently, environmental pollutants have attracted significant attention owing to their wide application in the construction of hybrid electrolysis systems that can achieve considerable environmental benefits and energy-saving H_2_ production. Moreover, the transformation of waste into treasures is realized when environmental pollutants such as formaldehyde [10,11], iodides [12], hydrogen sulfide [13], and monomers derived from waste microplastics [14] (including ethylene glycol [15,16,17,18,19,20,21,22,23,24,25,26,27,28,29,30,31], 1,4-butanediol [32], and 1,6-diaminohexane [33]) are utilized as AFs. Therefore, the hybrid strategy reshapes conventional water electrolysis and provides great opportunities to fulfill the multiple goals of hydrogen energy production, valuable chemical manufacturing, and environmental pollutant degradation.

Polylactic acid (PLA; (C_3_H_4_O_2_)_n_) is a classic daily polyester engineering plastic with a global annual output of approximately 4.59 billion tons in 2022, which is expected to exceed 2 million tons by 2027 [34] (Figure S1). PLA is a high-performance biodegradable polymer artificially synthesized from natural biomass. Methods for reforming PLA waste primarily rely on mechanical, chemical, and biological routes [35]. The deteriorated mechanical and physical properties of PLA result from laborious mechanical routes [36]. Biological depolymerization requires specific active enzymes that operate under appropriate conditions; however, this route has low economic feasibility owing to its ultra-slow rate and invaluable degradation products (e.g., CO_2_ and H_2_O) [37]. Recently, several reports have been published on the chemical recycling of PLA waste into valuable chemicals and hydrogen fuels under mild conditions using photo- [38,39,40,41], electro- [18,42,43], or even thermal catalysis [44]. The transition metals reported for upgrading PLA include CdS/MoS_2_ [38], Pt/TiO_2−x_ [39], CoP/CdS [40], Pd-CdS [41], IrO_x_ [42], CuCoO_2_ [18], PdNi [43], and α-Fe_2_O_3_ [44]. However, expensive metal catalysts are often required, which limits their practical application.

*Saccharomycetes* cells are a type of natural and edible ferment and are frequently used for fermentation in the wine, tea, and food manufacturing industries. They are globally recognized probiotics that can be used as inexpensive pharmaceuticals to strengthen the stomach and improve digestion. They have been used by our group as biological templates to effectively absorb metallic ions because of their high surface area and negative charge while serving as C, N, and P sources during this biocarbonization process for preparing various electrocatalysts [45,46,47,48,49,50].

In this study, we describe the electrolytic oxidation of PLA hydrolysates paired with efficient H_2_ generation on robust N, P-doped Cu-based nanowires supported on NF (N, P-doped CuO_x_ NW/NF). This integrated catalyst was expediently prepared from a Cu(OH)_2_ NW precursor via a solid-solid reaction using domestic *Saccharomycetes* cerevisiae as the C, N, and P sources. The nonmetallic elements N and P were obtained from dry *Saccharomycetes* without relying on the hazardous gases NH_3_ and PH_3_, which is consistent with our previous studies. The introduction of low-electronegativity nonmetallic-element (i.e., N and P) doping effectively promoted catalytic performance. In addition, the possible pathway of the lactate oxidation reaction (LOR) was explored using electrochemical measurements and hydrogen nuclear magnetic resonance (^1^H NMR). Oxidation-decarboxylation has been proposed to be more plausible than decarboxylation-oxidation. Compared with the OER, the LOR can save nearly 224 mV to output a current density of 30 mA/cm^2^. This Cu-based NW electrocatalyst can be recycled at least five times without any activity degradation. Acetate, but not O_2_, was produced in the anodic compartment with high selectivity and Faradaic efficiency of >89.3% for H_2_ production.

## 2. Results and Discussion

### 2.1. Material Fabrication and Characterization

**Figure 1:**
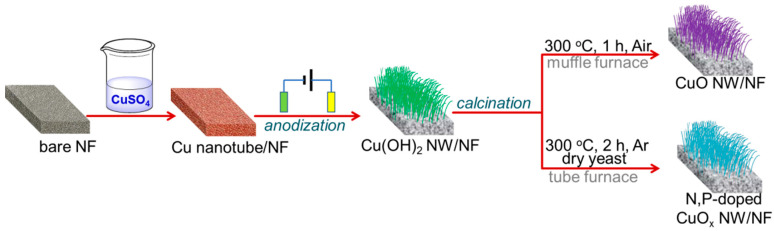
Schematic of the fabrication of Cu-based NW/NF.

Figure 1 shows the fabrication of the N, P-doped CuO_x_ NW/NF electrocatalyst using a facile three-step procedure. Initially, well-crystalline Cu(0) tubes were directly grown on a pre-cleaned 3D NF substrate via a spontaneous galvanic replacement reaction (Ni + Cu^2+^ → Ni^2+^ + Cu), during which the pristine NF was impregnated in concentrated CuSO_4_ solutions at room temperature. This replacement reaction is based on the fact that the standard redox potentials of Ni^2+/0^ and Cu^2+/0^ are −0.257 V and 0.342 V, respectively. Subsequently, the Cu tubes were transformed into Cu(OH)_2_ NW arrays via simple electrochemical oxidation. Finally, the Cu(OH)_2_ NW/NF precursor underwent calcination with *Saccharomycetes* at 300 °C in Ar gas flow to obtain the desired self-standing catalytic electrode. During annealing, the organic matter diffused from *Saccharomycetes* cells into the Cu-based NW. Additionally, CuO NW was prepared via direct calcination of Cu(OH)_2_ NW in air. The detailed synthetic procedure is described in Section 3. The commercial NF and Cu-based materials are shown in Figure S2, indicating that the color of the electrode changed from blue to black after calcination.

**Figure 2:**
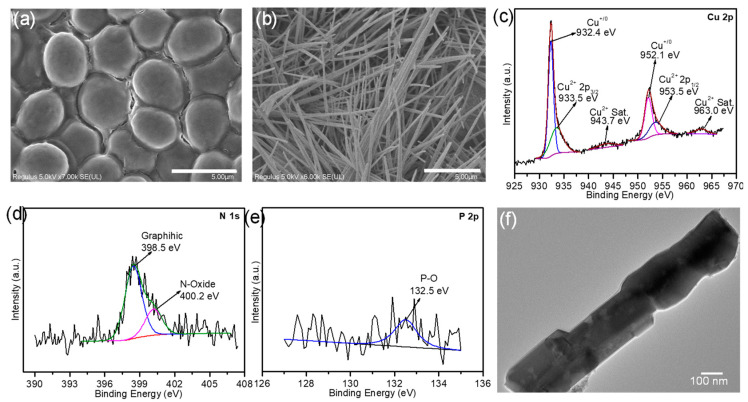
SEM images of (**a**) *Saccharomycetes* and (**b**) P, N-doped CuO_x_ NW/NF. High-resolution XPS spectra of (**c**) Cu 2p, (**d**) N 1s, and (**e**) P 2p of P, N-doped CuO_x_ NW. (**f**) High-resolution TEM image of the P, N-doped CuO_x_ NW.

The morphology of dry *Saccharomycetes* yeast powder was characterized using field-mission scanning electron microscopy (SEM). Pure microorganisms aggregate and microscale ellipses typically possess a smooth surface with an average diameter of approximately 4 μm (Figure 2a). Energy-dispersive X-ray spectroscopy (EDS) elemental mapping showed that the yeast was rich in several nonmetallic elements such as C, N, P, and O (Figure S3), which were distributed homogenously throughout the entire region and were beneficial for improving the catalytic performance of the electrocatalysts.

The morphologies of the as-prepared Cu-based nanomaterials were also characterized using SEM. The smooth surfaces of the NF were covered with dense micron-sized Cu cubes (Figure S4a,b), implying that metallic copper was rapidly deposited on the NF skeleton via a replacement reaction between Ni and Cu^2+^. The high-magnification SEM images in Figure S4c show that these Cu cubes were smoothly converted into Cu(OH)_2_ NW after anodization. These NW were well-aligned with a length of a few tens of micrometers and uniformly cross-grown over the surface of the NF substrate, endowing this composite with more accessible active sites and faster mass transport. As shown in Figure S4d, CuO maintained the morphological characteristics of the Cu(OH)_2_ NW precursor and became curved. After the Cu(OH)_2_ NW precursor underwent a solid-solid reaction with *Saccharomycetes*, the resulting N, P-doped CuO_x_ still exhibited the NW structure (Figure 2b and Figure S5). The crystal structures of the three Cu-based nanomaterials were investigated using X-ray diffraction (XRD) analysis. As illustrated in Figure S6, the XRD patterns of all samples contain three distinct peaks at 44.6°, 51.8°, and 76.3°, corresponding to the (111), (200), and (220) crystallographic planes of the Ni substrate. A set of weak diffraction peaks characteristic of Cu(OH)_2_ (JCPDS No. 35-0505) was observed, and the CuO cubes were transformed into Cu(OH)_2_ NW after anodic oxidation. The three diffraction peaks at 36.6°, 42.5°, and 61.5° for the two annealed products of Cu(OH)_2_ NW in the absence and presence of *Saccharomycetes* were assigned to the (111), (200), and (220) planes of the CuO phase (JCPDS No. 78-0428), respectively, confirming the successful preparation of CuO NW [51]. Moreover, Cu_3_P and Cu_3_N phases were not detected (Figure S7); therefore, N and P were confirmed to be doped into the CuO NW during the pyrolysis of *Saccharomycetes*.

The EDS elemental mapping images in Figure S8 quantitatively show that Cu, P, N, and O were homogeneously distributed throughout the N, P-doped CuO_x_ NW/NF. X-ray photoelectron spectroscopy (XPS) measurements were performed to determine the elemental composition and chemical valences. The XPS survey spectra shown in Figure S9a consistently confirm the presence of Cu, P, N, and O elements in the N, P-doped CuO_x_ NW. Figure 2c shows a high-resolution Cu 2p XPS spectrum. The two main peaks at 932.4 and 952.1 eV can be attributed to Cu^+^ or Cu(0) [52,53,54], implying that some divalent Cu in the original CuO NW was reduced by the volatile carbon species from *Saccharomycetes*. The two fitting peaks at 933.5 and 953.5 eV are consistent with the 2p_3/2_ and 2p_1/2_ binding energies of divalent Cu, respectively. Moreover, two evident satellite peaks at 943.7 and 963.0 eV reflect the presence of Cu^2+^. The N 1s peaks at 398.5 and 400.2 eV can be attributed to graphitic N and surface-oxidized N species, respectively (Figure 2d). The partial oxidation of the doped P element upon exposure to air was also confirmed by the P 2p XPS spectrum (Figure 2e). The O 1s XPS spectrum indicated the presence of two oxygen contributions related to the metal-oxygen and hydroxyl groups (Figure S9b). The high resolution of C 1s for N, P-doped CuO_x_ NW could be deconvoluted into four peaks centered at 284.6, 286.0, 287.7 and 289.3 eV, corresponding to the bonding states of C-C, C-O, C=O, and O-C=O, respectively (Figure S9c). The transmission electron microscopy (TEM) image of the material scraped from the resulting N, P-doped CuO_x_ NW/NF surface also shows the NW morphology with a diameter of approximately 300 nm (Figure 2f). In addition, no thin carbon layer was observed around the NW. The high-resolution TEM image shows a lattice spacing of 0.296 nm, corresponding to the (111) plane of copper oxide (Figure S10).

### 2.2. Electrochemical Oxidation of PLA Monomer Coupled with H_2_ Production

The process for upgrading PLA mainly comprises two steps: (i) hydrolysis of PLA, thermally catalyzed by NaOH, and (ii) electrolysis of the PLA lysate in NaOH, including sodium lactate 2e^−^ or 4e^−^ oxidation on the anode and the paired HER on the cathode. The PLA hydrolysate solution can be prepared using the alkaline hydrolysis method, which was performed with a PLA pellet in a 1.0 M NaOH solution at 125 °C for 14 h. The PLA polymer was completely digested into the sole monomer, i.e., lactate, according to the NMR test (Figure S11).

**Figure 3:**
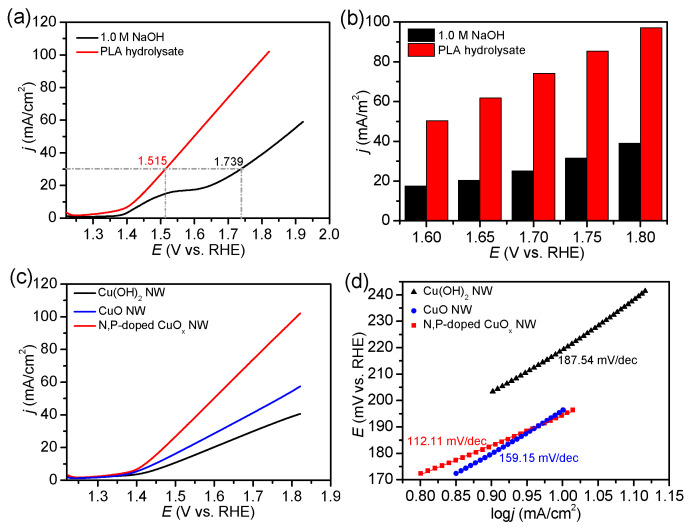
(**a**) LSV of N, P-doped CuO_x_ NW/NF in PLA hydrolysate and 1.0 M NaOH electrolyte. Scan rate: 5 mV s^−1^. (**b**) Comparison of the current densities of N, P-doped CuOx NW/NF at different potentials in 1.0 M NaOH (black) and PLA hydrolysate (red). (**c**) LSV of three Cu-based materials in the PLA hydrolysate. (**d**) Tafel plots with Cu-based electrodes for the LOR in PLA hydrolysate.

The catalytic ability of the three Cu-based NW materials toward the lactate oxidation reaction (LOR) was evaluated via electrochemical measurements in a typical three-electrode H-type configuration under alkaline conditions (Figure S12). A carbon rod, not a noble Pt plate, served as the counter electrode for HER. The linear sweep voltammetry (LSV) curve demonstrated that OER was triggered at approximately 1.60 V vs. RHE (the corresponding onset overpotential was 371 mV) using the N, P-doped CuO_x_ NW/NF as the working electrode in 1.0 M NaOH at pH 13.4 (Figure 3a). This indicated that the OER performance of the N, P-doped CuO_x_ NW/NF was relatively moderate; a broad pre-wave was observed at 1.50 V due to the oxidation of the NF substrate. A current density of 30 mA/cm^2^ was delivered at a potential of 1.515 V in the PLA hydrolysate, saving 224 mV compared to that for the OER under the same conditions. Our results are consistent with those of anodic catalysts used for other plastic-monomer-assisted H_2_ generation processes (Table S1). As shown in Figure 3b, a higher current density can be achieved via this hybrid electrolysis with LOR as the anodic reaction compared to pure water electrolysis at the same potential. Therefore, the LOR can be employed as a half-reaction to replace the OER and reduce the energy consumption of cathodic H_2_ production.

The electrocatalytic activities of the as-prepared Cu-based catalysts for the LOR were compared via LSV measurements under the same conditions (Figure 3c). Among all the electrodes, the N, P-doped CuO_x_ NW/NF exhibited optimal catalytic performance. Compared to those of other electrodes in PLA hydrolysate, the N, P-doped CuO_x_ NW/NF electrode exhibited a lower Tafel slope of 112.11 mV/dec (Figure 3d), which indicated its high intrinsic electrocatalytic capability and rapid reaction kinetics toward the LOR. To better understand the reason for this high activity, the electrochemical impedance spectra and Tafel slopes were then measured. The Nyquist plot of the N, P-doped CuO_x_ NW/NF electrode demonstrated the smallest semicircle radius, implying its rapid charge transfer kinetics during LOR (Figure S13a). To evaluate the electrochemically active surface areas, the double-layer capacitances (C_dl_) of the three Cu-based NW were tested using CV measurements as a function of the scan rate (Figure S14). As shown in Figure S13b, the calculated C_dl_ value (6.61 mF cm^−2^) for N, P-doped CuO_x_ NW/NF was determined to be higher than that for Cu(OH)_2_ NW and CuO NW, implying that it has abundant electrochemical active sites for the LOR.

**Figure 4:**
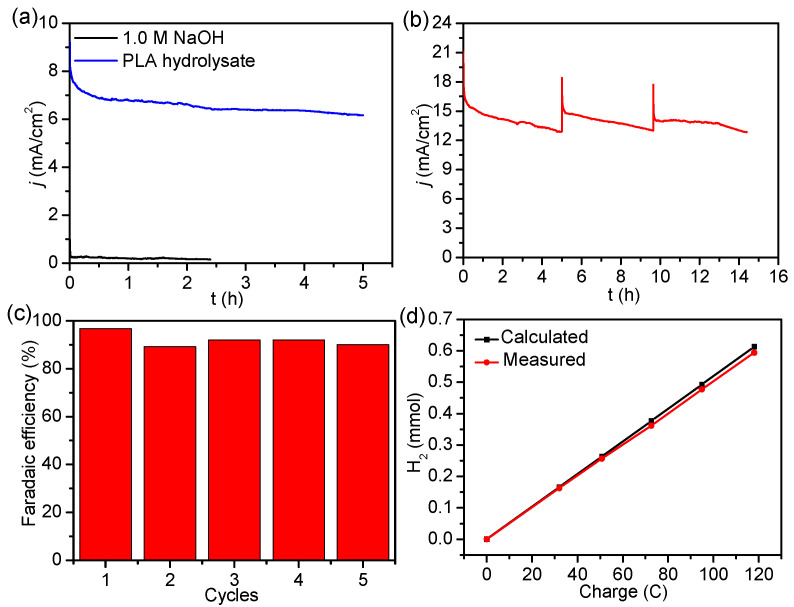
(**a**) Constant-potential electrolysis for the LOR at 1.42 V using N, P-doped CuO_x_ NW/NF in 1.0 M NaOH and PLA hydrolysate. (**b**) Current time curve of N, P-doped CuO_x_ NW/NF at 1.48 V with the intermittent addition of PLA hydrolysate. (**c**) Calculated Faradaic efficiencies for LOR in a sequence of five successive cycles. (**d**) Comparison of measured and theoretical amounts of H_2_.

Constant-potential electrolysis was conducted to investigate the stability of N, P-doped CuO_x_ NW/NF toward the LOR in PLA hydrolysate under vigorous stirring. As shown in Figure 4a, the delivered current experienced only a slight decrease during the 5 h of operation, which could be attributed to the gradual consumption of lactate in the electrolyte. In contrast, a negligible current was achieved using N, P-doped CuO_x_ NW/NF in pure NaOH without the addition of lactate. The data in Figure S15 show that the electrode maintained its original NW morphology after long-term electrolysis. The same electrode could be utilized in three electrolysis cycles with satisfactory stability toward the LOR (Figure 4b). High Faradaic efficiencies of >89.3% were obtained for H_2_ production during multiple electrolysis at 1.42 V (Figure 4c), demonstrating the high energy efficiency of this electrocatalytic system. According to a typical drainage method, a homemade device was built for the collection of H_2_ gas during the LOR at different electrolysis times (Figure S16). The measured H_2_ production was approximately equal to the calculated value (Figure 4d).

### 2.3. Electrochemical Mechanism of LOR

**Figure 5:**
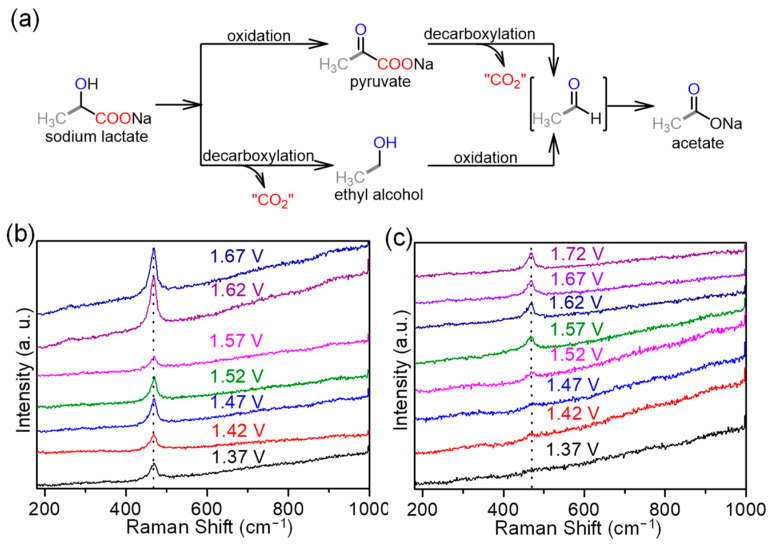
(**a**) Possible reaction pathway for the transformation of lactate into acetate. The removed CO_2_ species (i.e., “CO_2_”) may be released in the form of gas or further react with hydroxide ions to generate (bi)carbonate under basic conditions. Potential dependent in situ Raman spectra for N, P-doped CuO_x_ NW/NF collected under different applied potentials in 1.0 NaOH (**b**) and PLA hydrolysate (**c**).

To identify the oxidation product and possible intermediates of the LOR, ^1^H NMR spectroscopy was performed via electrolysis experiment in 1.0 M NaOH containing 0.1 M lactate. Product analyses were conducted at different stages of electrolysis. As shown in Figure S17, lactate was the only product of PLA hydrolysis, and acetate was determined to be the predominant product of the LOR with the as-prepared N, P-doped CuO_x_ NW/NF. Moreover, pyruvate was captured as the key intermediate during the LOR after electrolysis (0.5 h), which was due to the dehydrogenative transformation of hydroxyl groups into carbonyl groups. The pyruvate intermediate was then converted into the final acetate via decarboxylation. Ethyl alcohol was not detected in the NMR spectrum, suggesting that it was not a reaction intermediate. Acetate is sufficiently stable and can be used as an electrolyte for water splitting [5] because it does not undergo further decarboxylation. Based on these NMR results, oxidation-decarboxylation is proposed to be a more plausible reaction pathway than decarboxylation-oxidation for the LOR (Figure 5a).

To better gain mechanistic insight into this electro-driven LOR, LSV tests were conducted for the possible intermediate product of the LOR in 1.0 M NaOH (Figure S18). The catalytic onset potential of pyruvate is lower than those of ethyl alcohol, lactate, and acetate, suggesting that pyruvate is easily oxidized under the applied potential window. Although the oxidation profiles of lactate and ethyl alcohol were similar, ethyl alcohol was not detected by NMR spectroscopy (Figure S17). The oxidation of acetate requires substantially higher potentials, implying that the end product of the LOR could be acetate at the applied potentials. Subsequently, in situ Raman spectroscopy was performed to explore the active species on the N, P-doped CuO_x_ NW/NF toward the LOR. A featureless characteristic was observed at potentials less than 1.32 V in the NaOH blank solution (Figure S19). However, one distinct Raman band appeared at 468 cm^−1^ when the potential reached 1.37 V, and its intensity gradually increased with increasing applied potential (Figure 5b). This Raman peak is related to divalent Cu [55], which did not appear until the potential reached 1.52 V when the PLA hydrolysate was utilized as the electrolyte (Figure 5c). In the presence of lactate, the Raman peak of divalent Cu is interfered with by the LOR at potentials below 1.52 V [56]. Therefore, divalent Cu can be proposed as the active species on N, P-doped CuO_x_ NW/NF for the LOR.

## 3. Experimental Section

### 3.1. Fabrication of Cu Cubes/NF

The Cu cubes/NF were prepared using a facile method. The pre-cleaned NF was immersed into 50 mL of 0.1 M CuSO_4_ aqueous solution for 8 h in a water bath at 70 °C, followed by rinsing with a large amount of ultrapure water two times.

### 3.2. Preparation of Cu(OH)_2_ Nanowires/NF

A three-electrode system was set up for anodization, where the as-obtained Cu cubes/NF was used as the working electrode. A carbon rod, an Hg/HgO electrode, and a 2.0 M KOH solution were employed as the counter electrode, reference electrode, and electrolyte, respectively. The Cu(OH)_2_ nanowires/NF were prepared via chronopotentiometry (3000 s) under a current density of 6.0 mA cm^−2^.

### 3.3. Fabrication of CuO Nanowires/NF

The CuO nanowires/NF were made by directly heating the as-prepared Cu(OH)_2_ NW/NF in an air atmosphere in a muffle furnace at 300 °C for 1 h with a ramping rate of 5 °C/min.

### 3.4. Fabrication of P, N-Doped CuO Nanowires/NF

The obtained Cu(OH)_2_ NW/NF precursor (a size of 1 × 3 cm) was placed in a relatively small crucible, and then the whole crucible was placed in a large porcelain boat, and the other space in the large porcelain boat was filled with dry *Saccharomycetes* cerevisiae (weigh 5.0 g). Subsequently, the large porcelain boat was sealed with copper foil, placed in a tube furnace, thermally annealed at 300 °C for 10 h at a heating rate of 5 °C/min under an argon atmosphere, and then naturally cooled to room temperature. In this process, the active elements excited by *Saccharomycetes* cerevisiae chemically reacted with Cu(OH)_2_ NW/NF.

### 3.5. Material Characterization

Scanning electron microscopy (SEM) images afnd energy-dispersive X-ray (EDX) elemental mapping images were obtained using a JEOL JSM-6700F field emission scanning electron microscope (JEOL Ltd., Tokyo, Japan) equipped with an Oxford INCA X-sight energy-dispersive X-ray spectrometer. Powder X-ray diffraction (XRD) patterns were recorded using a Bruker D8 advanced X-ray diffractometer (Bruker, Billerica, MA, USA) with Cu K_α_ radiation (λ = 1.5418 Å) at a scan rate of 0.04°/s. The microscopic morphologies and structures were also characterized by high-resolution transmission electron microscopy (TEM, 200 kV) using a JEM-2100Plus microscope (JEOL Ltd., Tokyo, Japan). The Cu-based nanowire catalysts were removed from the NF substrate by sonication in absolute ethanol, and a drop of the mixture was dried on a microgrid copper network for TEM analysis. X-ray photoelectron spectroscopy (XPS) measurements were carried out using an AXIS SUPRA X-ray photoelectron spectrometer (Shimadzu, Kyoto, Japan) with monochromatized Mg Kα X-ray as the excitation source and C1s (284.60 eV) as the reference line. Nuclear magnetic resonance (NMR) spectra were recorded on Bruker AV 400 spectrometers at 400 MHz for ^1^H NMR using tetramethylsilane (TMS) as the internal standard. Gas chromatographic measurements (GC-2060F, Shandong Lunan Analytical Instruments, Ltd., ZaoZhuang, China) were conducted to quantify the amount of hydrogen gas produced.

### 3.6. Electrochemical Measurements

All electrochemical performance measurements were conducted with a CHI660D electrochemical workstation (Chenhua Co., Shanghai, China) at room temperature (25 ± 1 °C) using 1.0 M NaOH as the electrolyte. Cu-based nanowires supported on NF with a geometric area of ~1 cm^2^ were used as the working electrode; a carbon rod and Hg/HgO (1.0 M KOH) were utilized as the counter and reference electrodes, respectively, in a divided cell containing 1.0 M NaOH solution installed with a proton exchange membrane (N117). The scan rate for linear sweep voltammetry (LSV) was set to 10 mV s^−1^. Electrochemical impedance spectroscopy (EIS) was performed using alternating current impedance spectroscopy, with the working electrode biased at a suitable potential (e.g., 1.37 V vs. RHE for LOR), while the frequency ranged from 100 kHz to 0.01 Hz. Double-layer capacitances (C_dl_) were calculated by cyclic voltammetry in a potential window of 0.91–1.01 V vs. RHE with different scan rates. All polarization curves were iR compensated (100%) with regard to the ohmic resistance of the solution. Furthermore, the potentials were all converted to the RHE scale according to the Nernst equation {*E*(RHE) = *E*(Hg/HgO) + 0.059pH + 0.095, where *E*(RHE) is the converted potential versus RHE, and *E*(Hg/HgO) is the experimental potential measured against Hg/HgO}. Faradaic efficiencies (FE) for the production of H_2_ were calculated by the following equation: FE = (n × z × F/Q) × 100%, where n is the amount of hydrogen (mol), z is the number of electrons required to form a molecule of H_2_, Q is the quantity of electric charge (C), and F is the Faraday constant (96485 C mol^−1^).

### 3.7. In Situ Raman Measurements

The in situ electrochemical Raman spectroscopic measurements were conducted on a confocal Raman microscope, Renishaw’s Invia Reflex microscopy. The applied potential was controlled and adjusted using a CHI660 electrochemical workstation. All Raman spectra were acquired using a spectra-electrochemical quartz cell. In the measurements, N, P-doped CuO_x_ NW/NF served as the working electrode, an Hg/HgO electrode served as the reference electrode, and a Pt wire served as the counter electrode. The calibration was conducted with a silicon wafer at a wavenumber of 520 cm^−1^. The spectra after the *i*-t tests were collected in 1.0 M NaOH and PLA hydrolysate. The applied potential was in the range of 0.72 to 1.72 V vs. RHE, and the applied time was 120 s.

### 3.8. PLA Hydrolysis

Alkaline hydrolysis was carried out for depolymerization of PLA in 1.0 M NaOH, which is commonly used as the electrolyte for alkaline water electrolysis, and NaOH will be consumed during this step. Firstly, the PLA pellet (1.0 g) was washed with ethanol and deionized water, respectively. Then, the dried PLA was added to 20 mL of 1.0 M NaOH solution and transferred to a 50 mL Teflon-lined autoclave, which was sealed and maintained at 125 °C for 14 h to achieve the complete hydrolysis of the PLA polymer. After cooling to room temperature, the hydrolysate solution containing lactate (~0.7 M) was added to 0.56 g NaOH solid to restore the concentration of NaOH to 1.0 M. Then this hydrolysate was used as the electrolyte for electrochemical testing and analysis.

## 4. Conclusions

Herein, we describe free-standing N, P-doped CuO_x_ NW arrays on NF surfaces prepared by calcination using commercial yeast as a green and safe nitrogen and phosphorus source. This electrode can be used for the LOR in parallel with H_2_ production, achieving fast and mild upcycling of waste PLA to value-added C_2_ chemicals and hydrogen gas. Benefiting from the NW array structure and unique composition, the electrocatalyst exhibited high activity and robustness toward the LOR. In this new hybrid water electrolysis process, a large potential (224 mV) can be saved to deliver a current density of 30 mA/cm^2^ when the OER is replaced by the LOR. Importantly, pyruvate and acetate were identified as crucial intermediates and final products of LOR, and tandem oxidation-decarboxylation was proposed as a plausible pathway for LOR by NMR and electrochemical analyses. The catalytically active centers were found to be divalent Cu using in situ Raman techniques. The experimental results described herein provide new insights into the rational design of low-cost electrodes for hybrid water electrolysis using a monomer derived from waste plastics as the anodic feedstock.

## Data Availability

Data are contained within the article and Supplementary Materials.

## Supplementary Materials

The following supporting information can be downloaded at: https://www.mdpi.com/article/10.3390/molecules29225323/s1, Figure S1. Global production capacities of bioplastics 2022 (a) and 2027 (b); Figure S2. Optical photographs of the bare NF and as-prepared Cu-based materials supported on NF.; Figure S3. The measured SEM region (a) and elemental mappings (b~e) of *Saccharomycetes*.; Figure S4. SEM images of the bare NF (a), Cu tubes (b), Cu(OH)_2_ NW (c), and CuO NW (d).; Figure S5. Additional SEM images of P, N-doped CuO_x_ NW/NF at the same scale.; Figure S6. XRD pattern of CuO NW, Cu(OH)_2_ NW and P, N-doped CuO_x_ NW. For comparison, standard XRD patterns of Ni, Cu_2_O, CuO, and Cu(OH)_2_ are also provided.; Figure S7. Comparison for XRD patterns of N, P-doped CuOx NW, standard Cu_3_P and Cu_3_N.; Figure S8. The measured SEM region (a) and elemental mappings (b~g) of the P, N-doped CuO_x_ NW/NF.; Figure S9. The survey XPS spectra (a), high resolution XPS spectra of O 1s (b) and C 1s (c) of P, N-doped CuO_x_ NW.; Figure S10. HRTEM image of P, N-doped CuO_x_ NW.; Figure S11. ^1^H NMR test of the freeze-dried sample from PLA hydrolysate (D_2_O as the solvent).; Figure S12. The optical photograph of the electrolyzer for lactate oxidation paired with hydrogen production.; Figure S13. (a) Nyquist plots of different Cu-based catalysts at a constant potential of 1.372 V vs. RHE. (b) Plots of current density as a function of scan rate for different catalysts.; Figure S14. CV curves for Cu(OH)_2_ NW/NF (a), CuO NW/NF (b), and N, P-doped CuO_x_ NW (c) at scan rates from 5 to 25 mV/s in a potential range without competing Faradaic reaction.; Figure S15. SEM image of N, P-doped CuO_x_ NW/NF after long-term electrolysis in 1.0 M NaOH with PLA hydrolysate.; Figure S16. A homemade device for hydrogen gas collection during LOR at different electrolysis time.; Figure S17. ^1^H NMR spectroscopy of PLA hydrolysate (pink), 0.1 M lactate after electrolysis at 1.42 V vs. RHE for 0.5 h (green) and 8 h (navy). For comparison, the ^1^H NMR spectroscopy of commercial sodium acetate (blue), sodium pyruvate (red), sodium lactate (black) are also provided under similar test conditions.; Figure S18. LSV curve of N, P-doped CuO_x_ NW/NF in 1.0 M NaOH with adding 0.1 M sodium lactate and various possible intermediate products (sodium pyruvate, ethyl alcohol, and sodium acetate) during LOR.; Figure S19. Potential dependent in situ Raman spectra for N, P-doped CuO_x_ NW/NF collected under different applied potentials (0.72~1.32 V) in 1.0 NaOH.; Table S1. Performance comparison of anodic catalysts for hydrogen generation coupled with oxidation of plastic monomers^a^.
