# Supplementary material for: Electrochemical Upgrading of Waste Polylactic Acid Plastic for the Coproduction of C2 Chemicals and Green Hydrogen
Source: Molecules. 2024 Nov 12;29(22):5323. doi: 10.3390/molecules29225323 (PMC11596206; doi:10.3390/molecules29225323)
Supplement: Supplementary file 1 [file molecules-29-05323-s001.zip › molecules-3290600-supplementary.pdf]

Electrochemical Upgrading of Waste Polylactic Acid Plastic for the Coproduction of C_2 Chemicals and Green Hydrogen

Daili Xiang [1], Kexin Zhou [1], Jiahui Huang [1], Qing Kang [2], Hao Li [1,*], Yuhui Duan [1], Jialei Du [1,3,*] and Hong Liu [1,4,*]

1 Institute for Advanced Interdisciplinary Research (iAIR), Collaborative Innovation Center of Technology and Equipment for Biological Diagnosis and Therapy in Universities of Shandong, University of Jinan, Jinan 250022, China
2 School of Chemistry and Chemical Engineering, University of Jinan, Jinan 250022, China
3 Department of Chemistry, National University of Singapore, 3 Science Drive 3, Singapore 117543, Singapore
4 State Key Laboratory of Crystal Materials, Shandong University, Jinan 250100, China

* Correspondence: 202421100229@stu.ujn.edu.cn (H.L.); ifc_dujl@ujn.edu.cn (J.D.); hongliu@sdu.edu.cn (H.L.)

EXPERIMENTAL

Chemicals.

Dry yeast (*Saccharomycetes cerevisiae*) was purchased from Angel Yeast Co. Ltd. Sodium hydroxide (NaOH, 99%), sodium acetate (AcONa, 99%), acetic acid (AcOH, 99.5%), and ethanol (EtOH, 95%) were bought from China National Pharmaceutical Group Co. Polylactic acid (PLA, particle size: 3 mm, molecular weight: ~80000), deuterium water (D_2O, 99.9 atom % D), and pyruvic acid ($CH_3COCOOH$, 98%) were purchased from Shanghai Macklin Biochemical Co. Ltd. Nickel foam (NF, thickness: 1.0 mm, ~99.9%) was obtained from Kunshan Jiayisheng Electronics Co. Ltd. (Jiangsu, China). Before use, the NF (1 × 3 cm) was pretreated in 1 M HCl solution for 10 min to remove the surface oxide layer and ultrasonicated with water and ethanol for 3 min successively. The Nafion N117 membrane was purchased from Tianjin Goose Union Technology Co. Ltd. Before electrochemical tests, this membrane was pre-cleaned by boiling in 5% H_2O_2 (1 h) and then 0.1 M H_2SO_4 (1 h), and finally washed with H_2O (20 mL) to remove any possible contaminants. All other reagents were analytical grade and used as received. All electrolyte solutions were prepared with ultrapure water (18.2 MΩ·cm) unless stated otherwise.

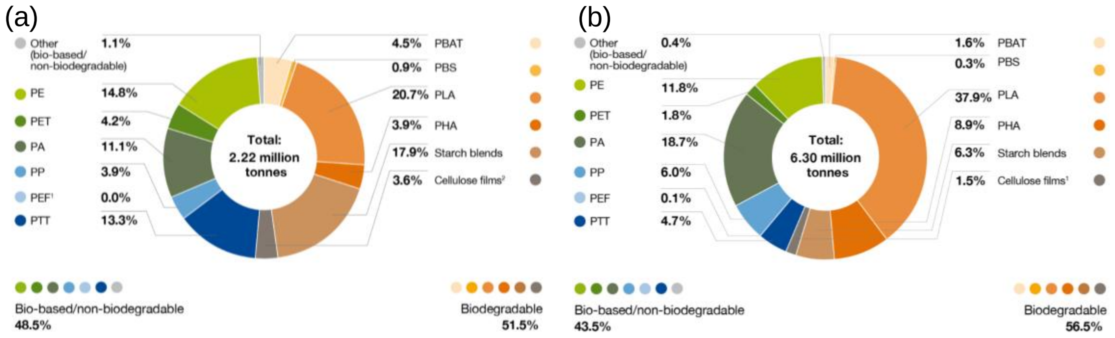

Figure S1. Global production capacities of bioplastics 2022 (a) and 2027 (b)

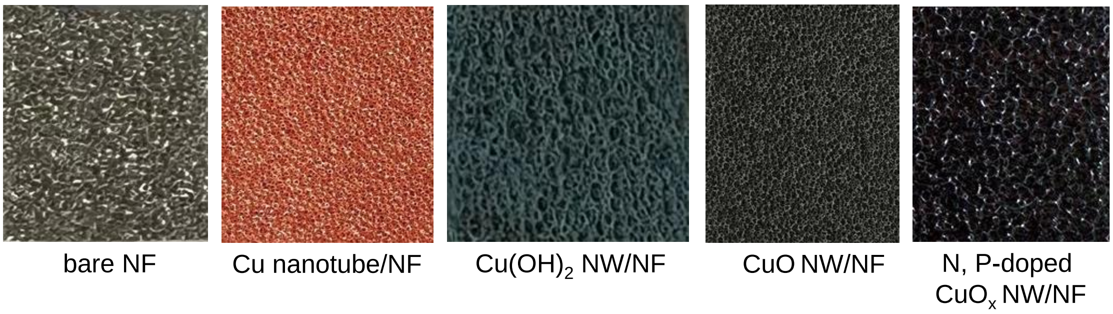

Figure S2. Optical photographs of the bare NF and as-prepared Cu-based materials supported on NF.

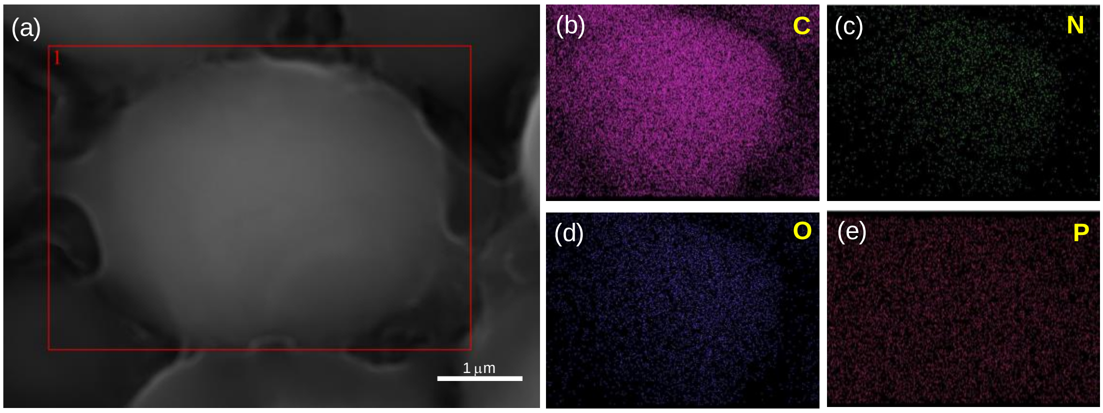

Figure S3. The measured SEM region (a) and elemental mappings (b~e) of *Saccharomycetes*.

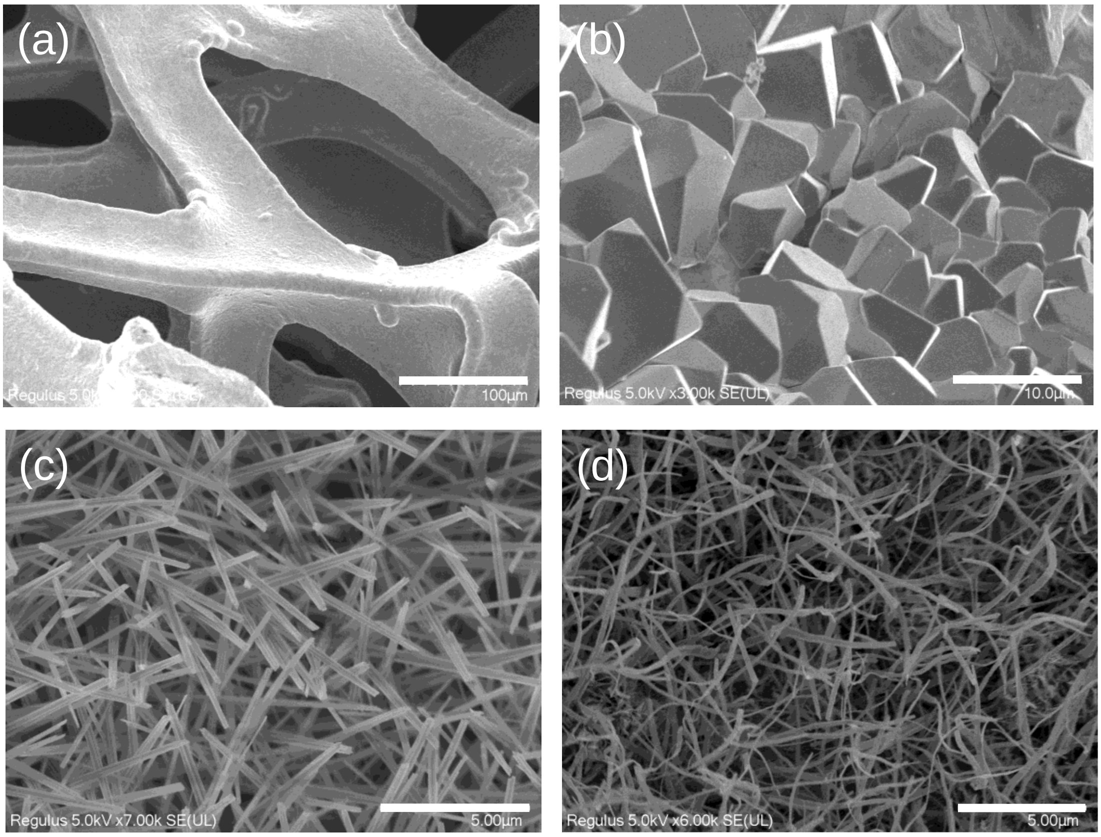

Figure S4. SEM images of the bare NF (a), Cu tubes (b), $Cu(OH)_2$ NW (c), and CuO NW (d).

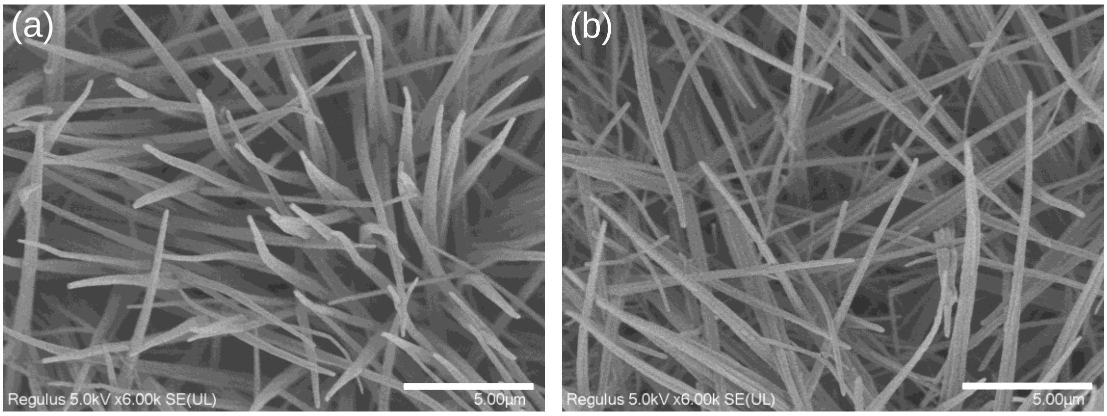

Figure S5. Additional SEM images of P, N-doped CuO_x NW/NF at the same scale.

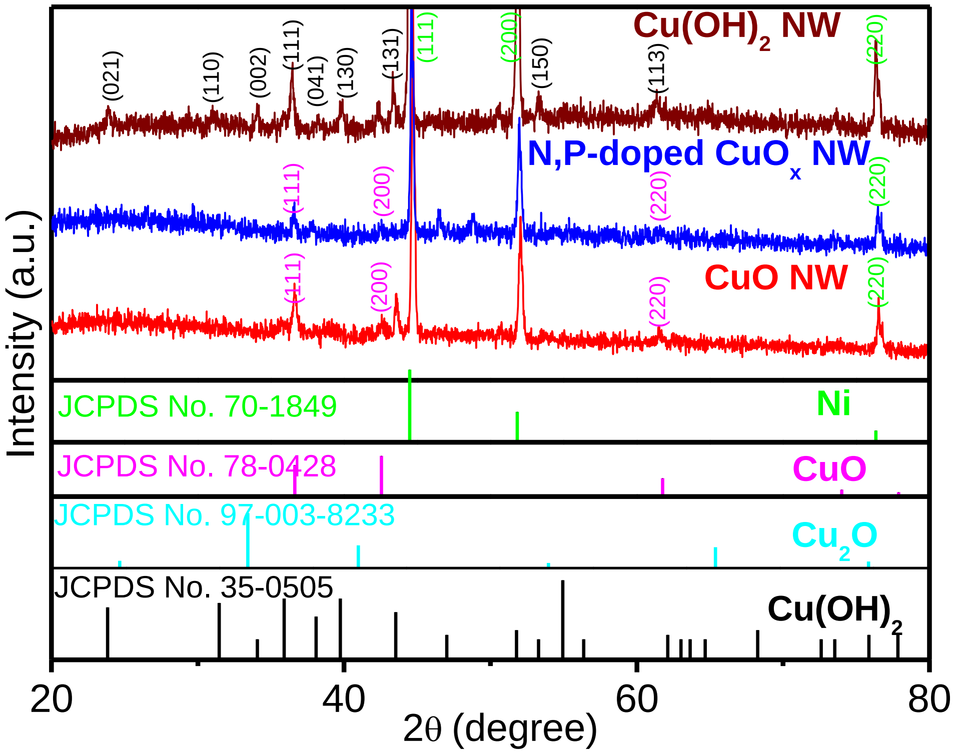

Figure S6. XRD pattern of CuO NW, $Cu(OH)_2$ NW and P, N-doped CuO_x NW. For comparison, standard XRD patterns of Ni, Cu_2O, CuO, and $Cu(OH)_2$ are also provided.

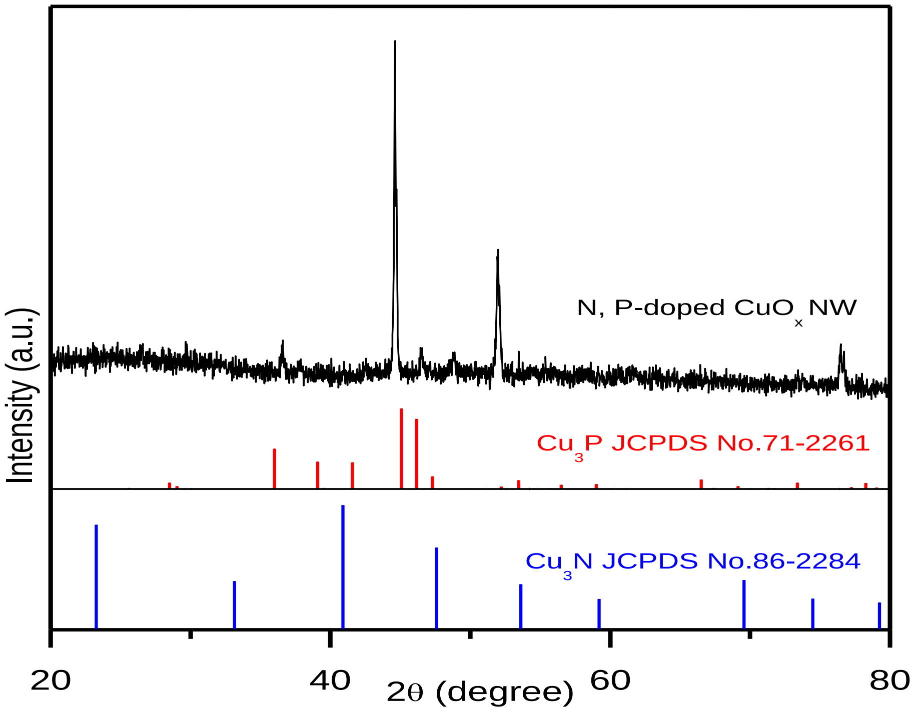

Figure S7. Comparison for XRD patterns of N, P-doped CuO_x NW, standard Cu_3P and Cu_3N.

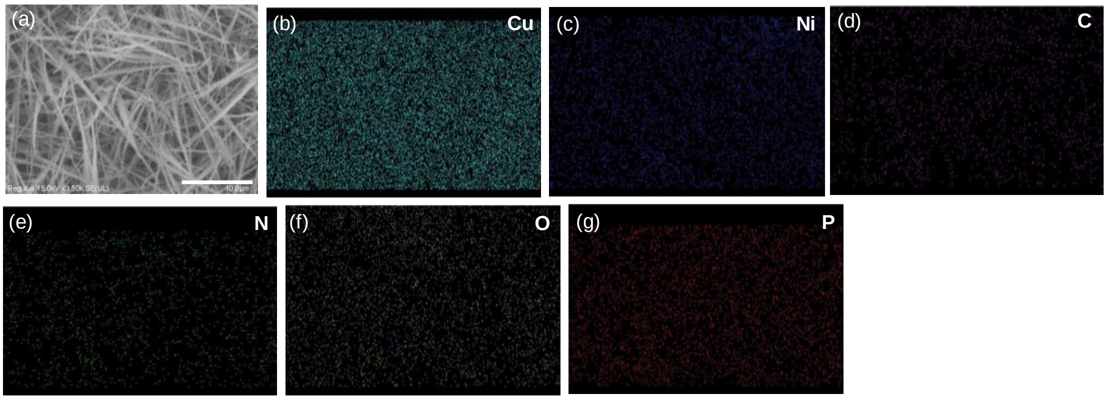

Figure S8. The measured SEM region (a) and elemental mappings (b~g) of the P, N-doped CuO_x NW/NF.

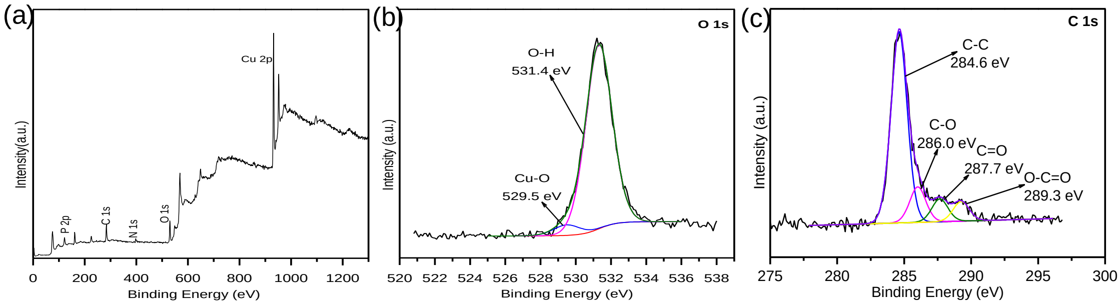

Figure S9. The survey XPS spectra (a), high resolution XPS spectra of O 1s (b) and C 1s (c) of P, N-doped CuO_x NW.

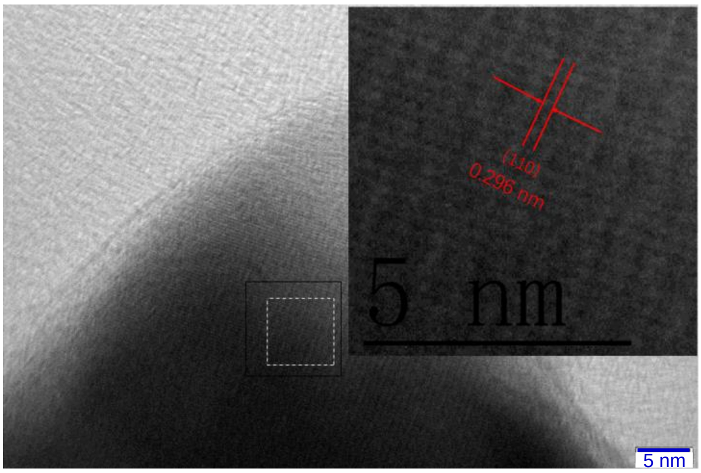

Figure S10. HRTEM image of P, N-doped CuO_x NW.

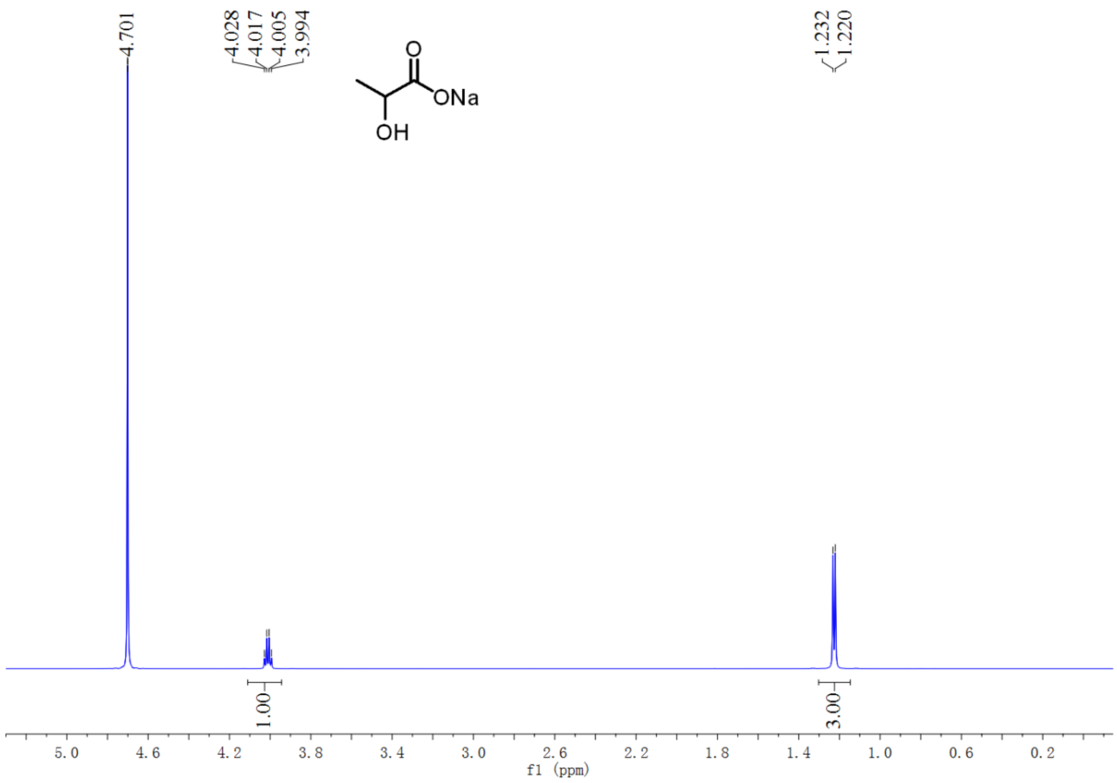

Figure S11. ^{1}H NMR test of the freeze-dried sample from PLA hydrolysate (D_2O as the solvent).

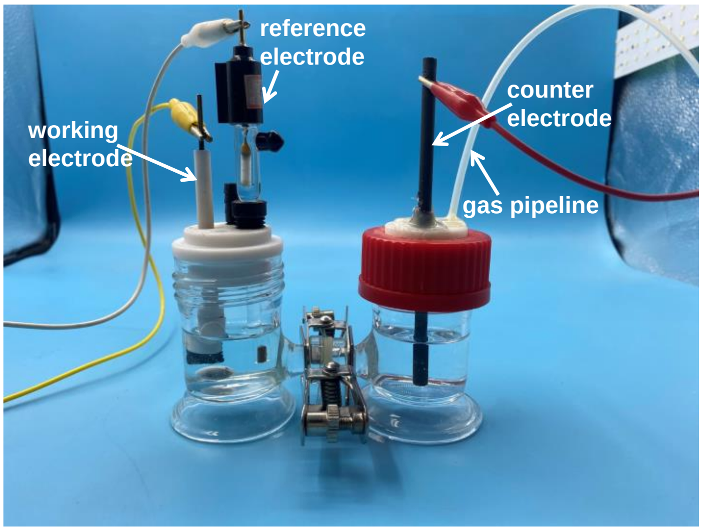

Figure S12. The optical photograph of the electrolyzer for lactate oxidation paired with hydrogen production.

Table S1. Performance comparison of anodic catalysts for hydrogen generation coupled with oxidation of plastic monomers[a]

Plastics	Oxidizable Monomer	Anode	Oxidation Products	E_1 (V)	E_2 (V)	ΔE (mV)	*J* (mA/cm^2)	Ref.
PET	1 M EG	Pd/NF-10	carbonate	1.69	~0.5[b]	1190	100	15[c]
PET	0.3 M EG	$CoNi_{0.25}P$/NF	formate	2.018	1.78	238	500	16
PET	0.5 M EG	Ni_1-CoP	formate	1.583	1.41	173	50	17
PET	EG	$CuCo_2O_4$/NF	formate	1.53	1.23	300	10	19
PET	EG	CuO	formate	1.64	1.38	260	10	18
PET	EG	Ni_3N/W_5N_4	formate	1.7	1.47	230	50	20[d]
PET	0.3 M EG	Ni_3N-$Ni_{0.2}Mo_{0.8}N$	formate	1.66	1.43	230	100	21
PET	0.17 M EG	$Mn_{0.1}Ni_{0.9}Co_2O_{4-\delta}$	formate	1.742	1.51	232	50	22
PET	0.1 M EG	Co-Ni_3N/CC	formate	1.83	1.46	370	50	23
PET	EG	Pd-NiTe/NF	formate	1.524	1.35	174	100	28
PET	0.1 M EG	Co,Cl-NiS/NF	formate	1.80	1.51	290	100	26[e]
PET	0.3 M EG	$Ni(OH)_2$/NF	formate	1.74	1.31	370	100	27[c]
PET	EG	$NiCu_{60s}$/NF	formate	1.71	1.45[b]	265.7	100	31
PET	1.0 M EG	Pd-$Ni(OH)_2$/NF	GA	1.65	0.69	960	100	25
PET	0.3 M EG	Pd-$CuCo_2O_4$/NF	GA	2.19	1.11	1080	200	29[f]
PET	0.1 M EG	Pt-$Ni(OH)_2$/NF	GA	1.57	0.69	880	100	30
PBT	0.1 M BDO	treated NF	succinate	1.57	1.36	210	50	32
PA-66	HMD	$Ni_3S_2@Fe_2O_3$	adiponitrile	~1.651[b]	1.46	191	250	33
PLA	lactate	N,P-doped CuO_x NW/NF	acetate	1.739	1.515	224	30	Our work[g]

[a]All the electrolyte is 1.0 M KOH unless otherwise specified. [b]This value is estimated according to the electrochemical measurements from the reference. [c]The electrolyte is 10 M KOH. [d]The electrolyte is 1.0 M KOH in seawater. [e]The electrolyte is 2.0 M KOH. [f]The electrolyte is 1.0 M NaOH + 0.5 M NaCl. [g]The electrolyte is 1.0 M NaOH. E_1 and E_2 represent the input voltages required for OER and the oxidation of plastic monomer vs. RHE, respectively. ΔE (*i.e.*, $E_1 - E_2$) represents the voltage saved when OER is replaced by the oxidation of plastic monomer. PET = polyethylene terephthalate, EG = ethylene glycol, PBT = polybutylene terephthalate, GA = glycolic acid, BDO = 1,4-butanediol, CC = carbon cloth, PA-66 = polyamide-66, HMD = hexamethylene diamine.

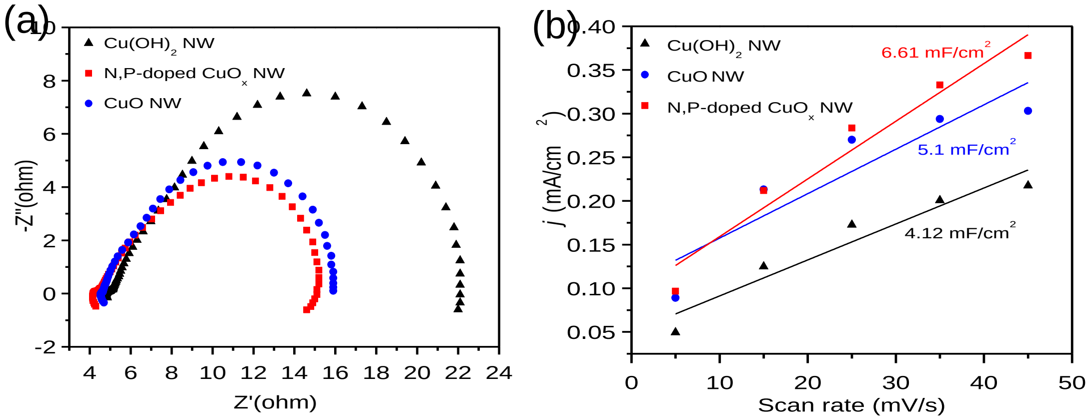

Figure S13. (a) Nyquist plots of different Cu-based catalysts at a constant potential of 1.372 V vs. RHE. (b) Plots of current density as a function of scan rate for different catalysts.

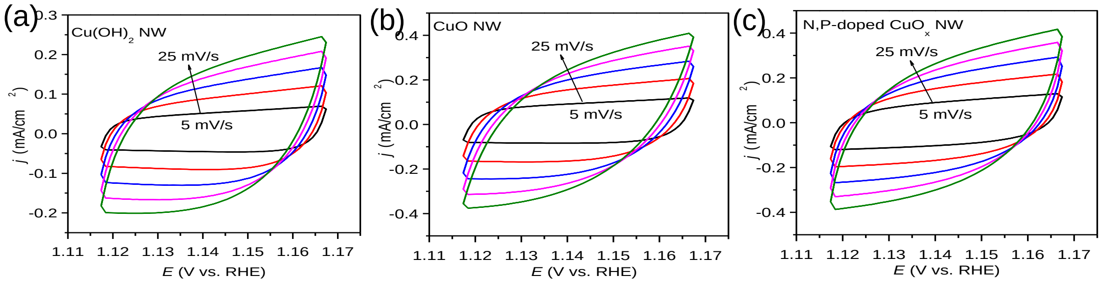

Figure S14. CV curves for $Cu(OH)_2$ NW/NF (a), CuO NW/NF (b), and N, P-doped CuO_x NW (c) at scan rates from 5 to 25 mV/s in a potential range without competing Faradaic reaction.

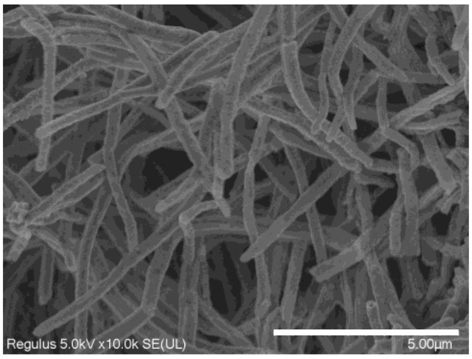

Figure S15. SEM image of N, P-doped CuO_x NW/NF after long-term electrolysis in 1.0 M NaOH with PLA hydrolysate.

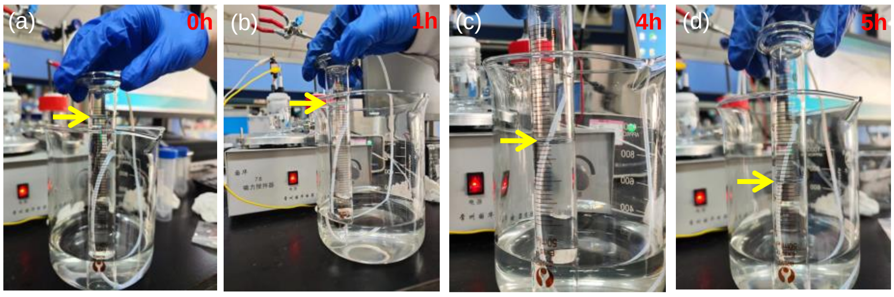

Figure S16. A homemade device for hydrogen gas collection during LOR at different electrolysis time.

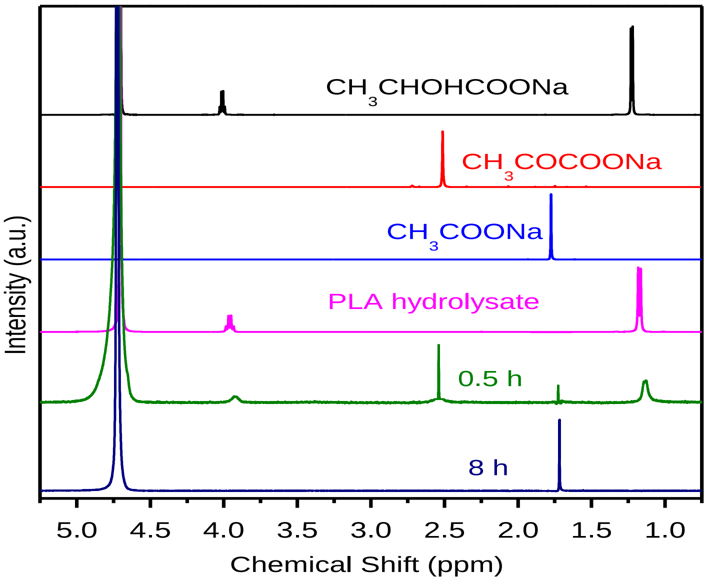

Figure S17. ^{1}H NMR spectroscopy of PLA hydrolysate (pink), 0.1 M lactate after electrolysis at 1.42 V vs. RHE for 0.5 h (green) and 8 h (navy). For comparison, the ^{1}H NMR spectroscopy of commercial sodium acetate (blue), sodium pyruvate (red), sodium lactate (black) are also provided under similar test conditions.

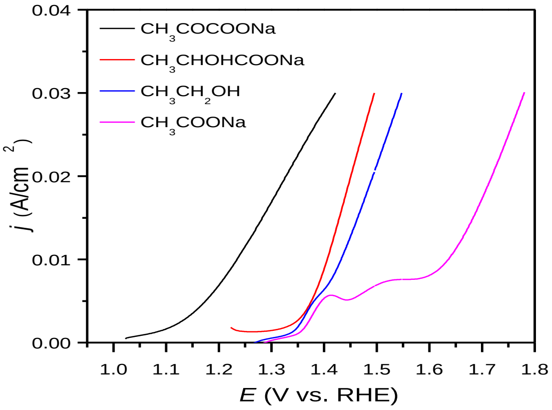

Figure S18. LSV curve of N, P-doped CuO_x NW/NF in 1.0 M NaOH with adding 0.1 M sodium lactate and various possible intermediate products (sodium pyruvate, ethyl alcohol, and sodium acetate) during LOR.

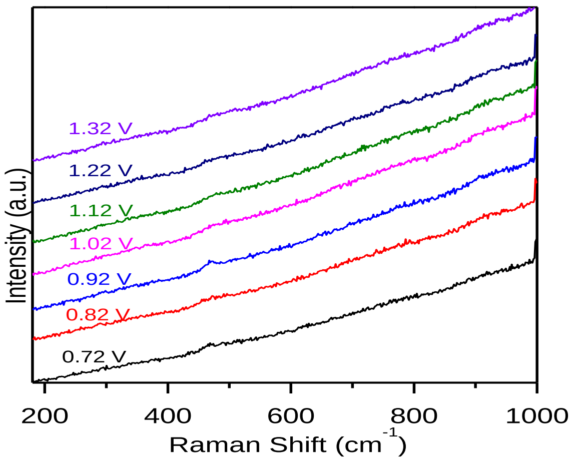

Figure S19. Potential dependent *in situ* Raman spectra for N, P-doped CuO_x NW/NF collected under different applied potentials (0.72~1.32 V) in 1.0 NaOH.
